# 

**Published:** 1845-03

**Authors:** 


### WESTERN JOURNAL

OF

### MEDICINE AND SURGERY.

Vol III, No III

### EDITORS.

---

DANIEL DRAKE, M. D.,

PROFESSOR OF PATHOLOGY AND THE PRACTICE OF MEDICINE IN THE MEDICAL INSTITUTE OF LOUISVILLE:

LUNSFORD P. YANDELL, M. D.,

PROFESSOR OF CHEMISTRY AND PHARMACY IN THE SAME:

THOMAS W. COLESCOTT, M. D.

### *Receipts of Medical Journal for February 1845.*

| | |
|---|---|
| Dr. W. H. Maury, Somerville, Tenn., - - | $5 00 |
| Dr. Wilson, Hebron, Ohio, - - - - - | 5 00 |
| Dr. Gault, City, - - - - - - | 10 00 |
| Dr. E. D. Wheeler, Murfreesboro, Tenn., - - - | 5 00 |
| Dr. Jackson, Gallatin, Mo., - - - - | 5 00 |
| Dr. C. A. Trumble, Chillicothe, Ohio, - - - | 5 00 |
| Dr. H. D. Coffield, Oxford, Ky., - - - - | 5 00 |
| Dr. J. W. Long, Shelbyville, Mo., - - - | 5 00 |
| Dr. J. Logue, Rural Hill, Tenn., - - - - | 5 00 |
| Dr. R. Steele, Paris, Ill., - - - - | 8 00 |
| Dr. Scales, Louisburg, Tenn, - - - - | 2 00 |
| Dr. J. W. Koen, Collinsville, Tenn., - - - | 15 00 |
| Dr. G. B. Harrison, Athens, Ky., - - . - | 5 00 |
| Dr. Askew, Chulahoma, Miss., - - - - | 5 00 |
| Dr. J. J. Murray, Montgomery Point, Ark., - - | 5 00 |
| Dr. N. Harris, Como, Miss., - - - - | 5 00 |

### TO SUBSCRIBERS.

We regret to find that, notwithstanding unusual expenditures upon the present volume of this Journal and its very material improvement, our subscribers have this year been more than usually remiss. We would give notice that all who may not remit promptly shall be forthwith and constantly harrassed with every form of dun until they are worried out. Their names shall appear in a black list on the back of the Journal, and, simultaneously, they shall be called upon and persecuted by duns of every kind, travelling agents, local agents, constables, sheriffs, and catchpoles. We are shamefully deprived of our dues and we mean to have justice done us.

PRENTICE & WEISSINGER.

☞Receipt by mail, at our risk, under frank of post-master or post-paid.

---

### TRAVELING AGENTS FOR THE WESTERN JOURNAI OF MEDICINE AND SURGERY.

JNO. F. JOHNSTON and JAS. H. STEVENSON, Kentucky—W. H. & W. M. WHEELER, Georgia, N. Carolina, S. Carolina, Alabama, and Arkansas—C. W. JAMES, Ohio, Indiana, and Michigan—GEO. C. BRETT, Tennessee—J. F. EASTON, Illinois and Missouri—WM. H. WELD & SON, Mississippi and Louisiana.

---

The WESTERN JOURNAL OF MEDICINE and SURGERY is published monthly by the undersigned, at the corner of Main and Fifth streets, Louisville, at $5 per annum, payable in advance. Each number contains 96 pages, making two volumes in the year of more than 550 pages.

Contributions to its pages by the Physicians of the Valley of the Mississippi, addressed to the Editors, are respectfully solicited.

Letters on business, to be addressed, postage paid, to the publishers.

☞Postmasters, by the regulations of the Postoffice Department, will frank letters containing subscription money, and all remittances so franked are at the risk of the publishers.

*January*, 1844. PRENTICE & WEISSINGER.

